# A network approach to mixing delegates at meetings

**DOI:** 10.7554/eLife.02273

**Published:** 2014-02-04

**Authors:** Federico Vaggi, Tommaso Schiavinotto, Jonathan LD Lawson, Anatole Chessel, James Dodgson, Marco Geymonat, Masamitsu Sato, Rafael Edgardo Carazo Salas, Attila Csikász-Nagy

**Affiliations:** Department of Computational Biology, Fondazione Edmund Mach, San Michele all’Adige, Italyfederico.vaggi@fmach.it; U-Hopper, Trento, Italy; the Gurdon Institute and the Genetics Department, University of Cambridge, Cambridge, United Kingdom; the Gurdon Institute and the Genetics Department, University of Cambridge, Cambridge, United Kingdom; the Gurdon Institute and the Genetics Department, University of Cambridge, Cambridge, United Kingdom; the Gurdon Institute and the Genetics Department, University of Cambridge, Cambridge, United Kingdom; Department of Life Science and Medical Bioscience, Waseda University, Tokyo, Japan; the Gurdon Institute and the Genetics Department, University of Cambridge, Cambridge, United Kingdomcre20@cam.ac.uk; Department of Computational Biology, Fondazione Edmund Mach, San Michele all’Adige, Italyattila.csikasz-nagy@fmach.it; the Randall Division of Cell and Molecular Biophysics and Institute of Mathematical and Molecular Biomedicine, King’s College London, London, United Kingdom

**Keywords:** cutting edge, meeting, interdisciplinary research, collaboration, social network, graph theory

## Abstract

Delegates at scientific meetings can come from diverse backgrounds and use very different methods in their research. Promoting interactions between these ‘distant’ delegates is challenging but such interactions could lead to novel interdisciplinary collaborations and unexpected breakthroughs. We have developed a network-based ‘speed dating’ approach that allows us to initiate such distant interactions by pairing every delegate with another delegate who might be of interest to them, but whom they might never have encountered otherwise. Here we describe our approach and its algorithmic implementation.

Scientists have been going to conferences for more than 450 years, but the basic format of talks followed by questions–with regular breaks for informal interactions over a drink or a meal–has remained largely the same. It is possible to foresee many ways in which conferences may evolve in the years ahead, but the main attraction is likely to remain the opportunity for scientists to meet and network, to develop ideas and collaborations, and to drink large amounts of tea, coffee and alcohol.

Compared to their 16th century ancestors, modern conferences are more inclusive than they have ever been, with diverse selections of delegates and speakers from around the world. However, the Q & A sessions after talks offer only limited opportunities for meaningful speaker-audience dialogue (Moore, 2010), and the discussion is often dominated by the senior scientists among the delegates. Although coffee breaks–the most productive part of scientific conferences (Obris, 2008; Stobbe et al., 2013)—provide more junior scientists with an opportunity to network, there is plenty of scope for improving the level of interactions between the senior and junior delegates.

Thus, current conference formats pose two key, interrelated problems: ‘breaking the ice’ (i.e., making it easy for younger scientists to introduce themselves, and ‘breaking the heat’ (i.e., discouraging the key players from only networking with their peers, thereby excluding their junior colleagues). There is a third problem at interdisciplinary conferences due to a lack of natural topics of conversation between people working in very different areas of research, so like tends to stick with like.

## Mixing delegates in a mutually interesting and novel way

Over the years conference organisers have tried many approaches to improve this state of affairs. There have been attempts at using web-based technologies to improve the interaction between the audience and the speakers, and to make social mixing easier and less intimidating. People have previously trialled virtual conferences, real-time Twitter feedback, and other web 2.0 technologies (Ebner and Reinhardt, 2009; Rigutto, 2013). These approaches have been successful in some fields, particularly related to media and journalism (Briggs, 2007; Picard, 2009), but they come with their own set of problems (Boyd, 2009) not the least a lack of technical know-how in the audience, particularly in the more academic fields.

There are also approaches to facilitate the formation of interest groups at conferences (Canning et al., 2013) and to help researchers identify potential collaborators from the web (Schall, 2013). The Mathematisches Forschungsinstitut Oberwolfach in Germany organizes weekly meetings where each participant has a napkin holder with her/his name on it, and at each meal the napkin holders are randomly distributed to each seat in the dining hall. Inspired by this, we recently developed an alternative, more ‘engineered’ approach to encouraging interactions at a meeting entitled Cell Polarity in the Systems Medicine Era, that was attended by 40 people. We call this approach interdisciplinary speed dating.

This was a satellite meeting that was held immediately after a larger meeting we organised at the Royal Society in London on the topic of cell polarity. In the smaller gathering the idea was to discuss in depth several topics brought up at the larger meeting in a more intimate setting, so all the delegates (a mix of junior group leaders, senior lab heads, and postdocs) were accommodated in the same building (at Chicheley Hall, which is about 50 miles north of London) to spend two days discussing science. Since the meeting was about cell polarity, the attendees consisted of a heterogeneous group of biophysicists, mathematical modellers, biochemists, in vivo researchers, and bioinformatics researchers. The combination of wide-ranging interests among the delegates, as well as the relatively small number of people attending, made this meeting an excellent test bed for our unorthodox conference approach.

**Figure 1.**
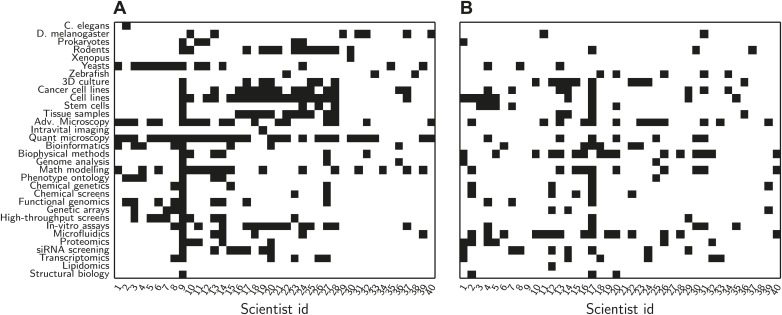
Data from initial survey sent to delegates. Prior to the meeting, we asked each delegate to choose, from a predetermined list, which methods they were familiar with, and which methods they wanted to learn more about. (**A**) Methods known by delegate. Each row represents a method and each column represents an individual delegate (names omitted for reasons of confidentiality). Delegates were most familiar with microscopy (both quantitative and advanced), cells (both cancer and stem) and mathematical modelling. This information was used to calculate the knowledge distance between delegates in Figure 3. (**B**) Methods that delegates wanted to learn more about. The most popular methods were biophysical methods and microfluidic devices.

To prepare for the satellite meeting we asked all the delegates to complete a short survey and eventually received responses from all but one delegate. In the survey, we asked the participants to state which methods they were familiar with from a list of 32 relevant methods, and to state which methods they wanted to learn more about (Figure 1). There was a wide variation between the number of methods known by the different delegates (ranging from 1 to 26, out of a total of 32; mean = 6.6, standard deviation = 4.4).

**Figure 2.**
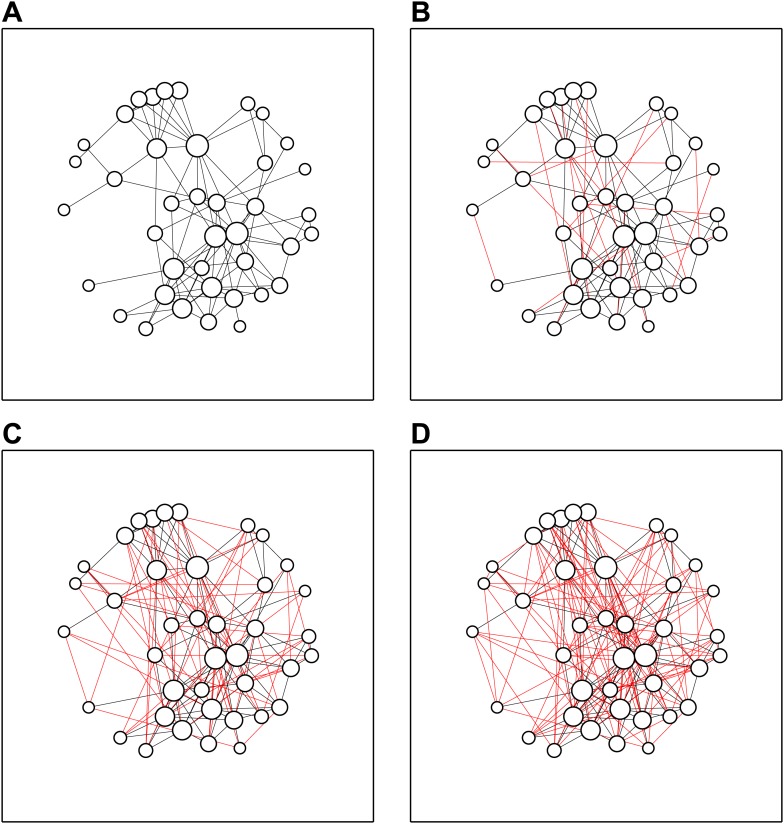
Speed dates increase network density. (**A**) The collaboration network before the meeting: some delegates already knew 10 or more other delegates, whereas others knew just one or two. (**B**) After the first round of speed dates, 20 new connections (shown in red) had been added to the network. (**C**, **D**) The network after three (**C**) and five (**D**) rounds of speed dates; α = 0.9.

Next, we asked which of the other delegates they had previously collaborated with. Interestingly, only about half of the relationships were mutual (51%)—this difference is probably due to some delegates only counting scientists with whom they have published papers, and other delegates favouring a less formal definition. However, it was notable that the delegates formed a connected network, with every delegate having collaborated with at least one other delegate (Figure 2A). We did not observe any statistically significant links between the gender of the scientist (13 female and 27 male scientists attended) and the extent of methodological knowledge or collaboration experience.

## At the conference

Over the two days of the conference, we dedicated two 90 minute sessions to speed dating. The first session consisted of five rounds of ice breakers in which each delegate was paired with another delegate with whom they had little in common (based on the methods they knew about and the people they had collaborated with). The intention was to cause the delegates to meet new and different people. Each round lasted 15 minutes, giving enough time for scientists to discuss their own work while still keeping the meetings brief and to the point. The atmosphere of the meetings was highly informal–coffee and refreshments were provided, and scientists had the choice to sit down at small tables or simply walk around near posters and discuss each other’s results.

**Figure 3.**
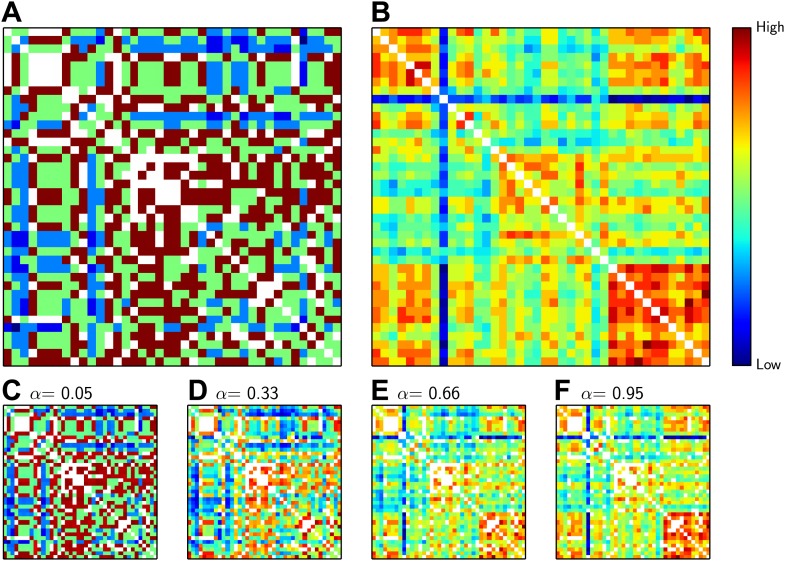
The distance between delegates. (**A**) Matrix showing the inverse of the ‘acquaintance distance’ between all pairs of delegates, who are arranged horizontally and vertically as in Figure 1. (**B**) Matrix showing the ‘knowledge similarity’ between all pairs of delegates: similarity increases with the number of methods known in common by both candidates, and decreases with the number of methods that are only known by one. The inverse acquaintance distance (**A**) and the knowledge similarity (**B**) have both been normalized to have zero mean and unit variance so that they can be added together, suitably weighted by α. (**C**–**F**) The sum of inverse acquaintance distance and the knowledge similarity for pairs of delegates for different values of α. Colder colours such as blue indicate low similarity and high distance (see colour bar); hotter colours such as red and brown indicate the reverse; white squares not on the diagonal represent delegates who have previously collaborated. The similarity measure that we used (the Rogers and Tanimoto similarity measure) penalized pairs with low overlap, which led to the pronounced vertical and horizontal blue lines observed for the delegate familiar with 26 methods in **B**.

In these five encounters, we used two criteria to match the delegates (Figure 3). The first criterion was ‘acquaintance distance’—the number of steps between the delegates on the collaboration graph based on their shared previous acquaintances. The second criterion was the ‘knowledge similarity’ between the two delegates in a pair–this number gave us a measure of how much knowledge the delegates had in common. We then added the inverse of the ‘acquaintance distance’ to the ‘knowledge similarity’. We used a parameter α to determine how much weight to give to each criterion in the sum (Figure 3C–F): α = 0 meant that only ‘acquaintance distance’ was considered when matching the delegates; α = 0.5 meant that ‘acquaintance distance’ and ‘knowledge similarity’ were considered equally; and α = 1 meant that only ‘knowledge similarity’ was considered. Finally, pairings between delegates who had previously collaborated—that is, whose acquaintance distance was equal to 1—were forbidden.

For each round, we sought to find the 20 pairs that would minimize the sum of the two values. In other words, we wanted to form pairs of delegates who were far away from each other in the network and who had minimal overlap in their knowledge. This means that we were looking for 20 deep blue squares in the matrices in Figure 3, subject to the constraint that we had to select one square (i.e., one delegate) from each column and from each row per round. This is a well-known problem in combinatorial optimization, and is called the Maximum-Weight Perfect Matching Problem (Edmonds, 1965); several polynomial-time exact algorithms exist for solving this problem (Gabow and Tarjan, 1991). Interestingly, the number of one-way collaborations (where one delegate reports a collaboration but the other does not) was significantly correlated with the number of methods known (r = 0.38, p-value = 0.015).

After the pairings for one round had been solved, we added a further constraint to prevent the same pairs being selected again in future rounds. Importantly, for each round we were solving for the optimal pairs which simultaneously maximized new knowledge gained for all scientists. This was a greedy strategy: choices made in early rounds introduced constraints (by reducing the number of possible pairs), which may prevent the algorithm from finding a best solution for later rounds. Hence a future improvement of this approach could be to modify the above implementation to maximise the sum of the distances across all rounds, instead of finding the optimal match on a per-round basis. In this manner, the solution would not need to decay through successive selection rounds.

**Figure 4.**
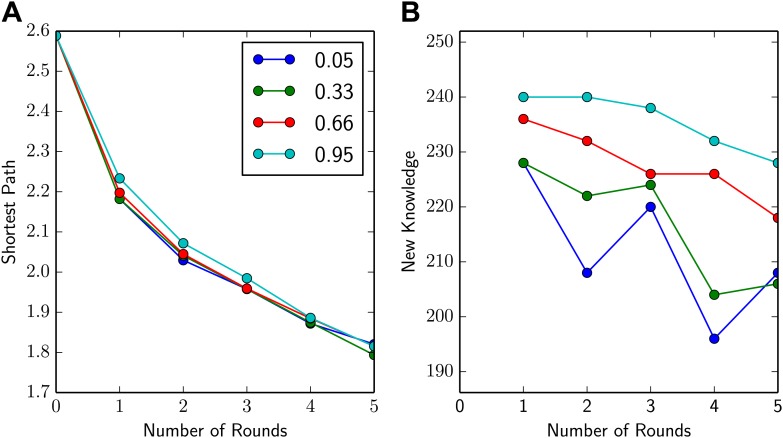
Increasing α increases new knowledge gained. (**A** and **B**) Average shortest path and new knowledge gained (i.e., the total number of new methods delegates were told about in a given round) vs number of rounds of speed dating for four different values of α. The shortest average path (**A**) fell quickly with the number of rounds of speed dating, almost irrespective of the value of α. However, new knowledge gained (**B**) increased with the value of α: this is to be expected because higher values of α mean that delegates are more likely to be paired with someone who can tell them about new methods.

To select an appropriate value of α, we simulated what would happen during five rounds of speed dating with four different values of this number. Since the meeting was quite small and the collaboration network was already quite dense to begin with, the average shortest path decreased quite rapidly, almost irrespective of the value of α (Figure 4A). However, the amount of new knowledge gained increased with the value of α (Figure 4B), so we used α = 0.9 when calculating pairs.

While the goal of the first five encounters was to match people who would not traditionally meet, the goal of the second round of encounters was to optimise the way people meet collaborators at conferences. Therefore, in the second round we sought to match delegates who were expert in particular methods with delegates wanting to learn about those methods (based on the data in Figure 1B). We did this by computing a ‘matching knowledge distance’, which quantified how much the methods known by one participant resembled the methods sought by another and vice-versa. We then summed the inverse of the ‘matching knowledge distance’ with the ‘acquaintances distance’, again weighted by a parameter α, and sought to maximise this sum. This was a popular approach, with a large proportion (>50%) of the people who responded to the post-meeting questionaire indicating that this part of the meeting led to the establishment of new collaborations.

The code we used to generate the pairs for this paper is available on the github repository: https://github.com/FedericoV/conference_pairings. When using our approach for very large conferences, it will be important to investigate the use of heuristics that allow the calculation of near-optimal solutions in a fraction of the time, as the current exact algorithm has O(N^3) scaling (where N is the number of delegates at the meeting).

## Conclusions and perspectives

When tweaking a successful idea, it is important to stay true to the original spirit and purpose one had in mind. Trying to improve scientific conferences, we sought to engineer a better way for scientists to become exposed to new ideas and unlikely conversation partners, meet potential collaborators, and learn more about specific methods they fancied. While our approach was decidedly unorthodox, our first attempt to implement it turned out much better than we expected: of the 24 delegates who commented on it in the post-meeting questionnaire, 21 (87.5%) were very enthusiastic and complimentary, and 12 mentioned that potential new collaborations were emerging from discussions at the meeting.

Of course there is room for improvement. For example, quite a few of the delegates at our meeting were lab heads, so at larger meetings attended by a lot of junior scientists it would be interesting to use ‘scientific seniority’ as an additional criterion when calculating the distance between delegates with a view to pairing these junior scientists with senior figures in the field. Alternatively, everyone attending the meeting could be asked what they hope to get out of the meeting (e.g., to meet new people, to learn more about various methods, to meet lab heads who are recruiting, and so on), and then one could attempt to match everyone according to their own stated aims. We are eager to explore these and other applications of our approach in the future.

## Notes

Rafael E Carazo-Salas and Attila Csikász-Nagy contributed equally to this work.
